# Association between periodontitis and disc structural failures in patients with cervical degenerative disorders

**DOI:** 10.1186/s13018-023-04381-5

**Published:** 2023-11-20

**Authors:** Xiaolong Chen, Dong Xue, Peng Cui, Ying Zhao, Shibao Lu

**Affiliations:** 1 Department of Orthopaedics, Xuanwu Hospital Capital Medical University, No.45 Changchun Street, Xicheng District, Beijing, 100053 China; 2 Department of Stomatology, Xuanwu Hospital Capital Medical University, No.45 Changchun Street, Xicheng District, Beijing, 100053 China

**Keywords:** Cervical spine, Disc degeneration, Endplate change, Periodontitis

## Abstract

**Objective:**

Recent studies have shown that the mouth–gut–disc axis may play a key role in the process of disc structural failures (including intervertebral disc degeneration (IDD) and endplate change) in the cervical spine and neck pain. However, the potential mechanisms underlying the mouth–gut–disc axis remain elusive. Therefore, we explored whether periodontal disease is associated with disc structural failures in patients with cervical degeneration disorders and clinical outcomes.

**Methods:**

Adults (aged > 18 years) who met open surgery criteria for cervical spine were enrolled in this prospective cohort study. Participants were allocated into two groups based on periodontal examinations before surgery: no/mild periodontitis group and moderate/severe periodontitis group. Data were evaluated using an independent t test and Pearson’s correlation analysis.

**Results:**

A total of 108 patients were enrolled, including 68 patients in the no/mild periodontitis group and 40 patients in the moderate/severe periodontitis group. The number of common causes of missing teeth (*P* = 0.005), plaque index (PLI) (*P* = 0.003), bleeding index (BI) (*P* = 0.000), and probing depth (PD) (*P* = 0.000) significantly differed between the two groups. The incidence rate of endplate change (*P* = 0.005) was higher in the moderate/severe periodontitis group than in the no/mild periodontitis group. A moderate negative association was found between the neck disability index (NDI) score and periodontal parameters (PLI: *r* = − 0.337, *P* = 0.013; BI: *r* = − 0.426, *P* = 0.001; PD: *r* = − 0.346, *r* = − 0.010).

**Conclusions:**

This is the first study to provide evidence that severe periodontitis is associated with a higher occurrence rate of disc structural failures and poor clinical outcomes in patients with cervical degenerative disorders.

## Introduction

Chronic neck pain is a significant public health problem, leading to disability and productivity loss worldwide [1, 2], especially in patients with cervical degenerative disorders. Disc structural failures such as intervertebral disc degeneration (IDD) and endplate change are regarded as the most common contributors to chronic neck pain [3–5]. Radiographic changes have been reported as the main criteria for evaluating disc structural failures, including scoring systems for IDD and endplate change developed by Pfirrmann et al. [5] and Modic et al. [6], respectively.

Inflammation is thought to be one of the main triggers for developing disc structural failure. Other potential factors for triggering disc structural failure include natural history, aging, smoking, mechanical and genetic factors, obesity, and inadequate metabolite transport. Growing evidence supports that infection of the intervertebral disc by low-virulence organisms may be crucial to the inflammatory response [7]. Our previous review reported a significant relationship between low-virulence organisms in IDD and endplate changes [7]. A further review demonstrated that the mouth–gut–disc axis (such as microbiome oral dysbiosis, gastrointestinal system, and intervertebral disc (IVD)) was potentially correlated with disc structural failure and spinal pain [8]. However, this hypothesis remains unclear.

**Fig. 1:**
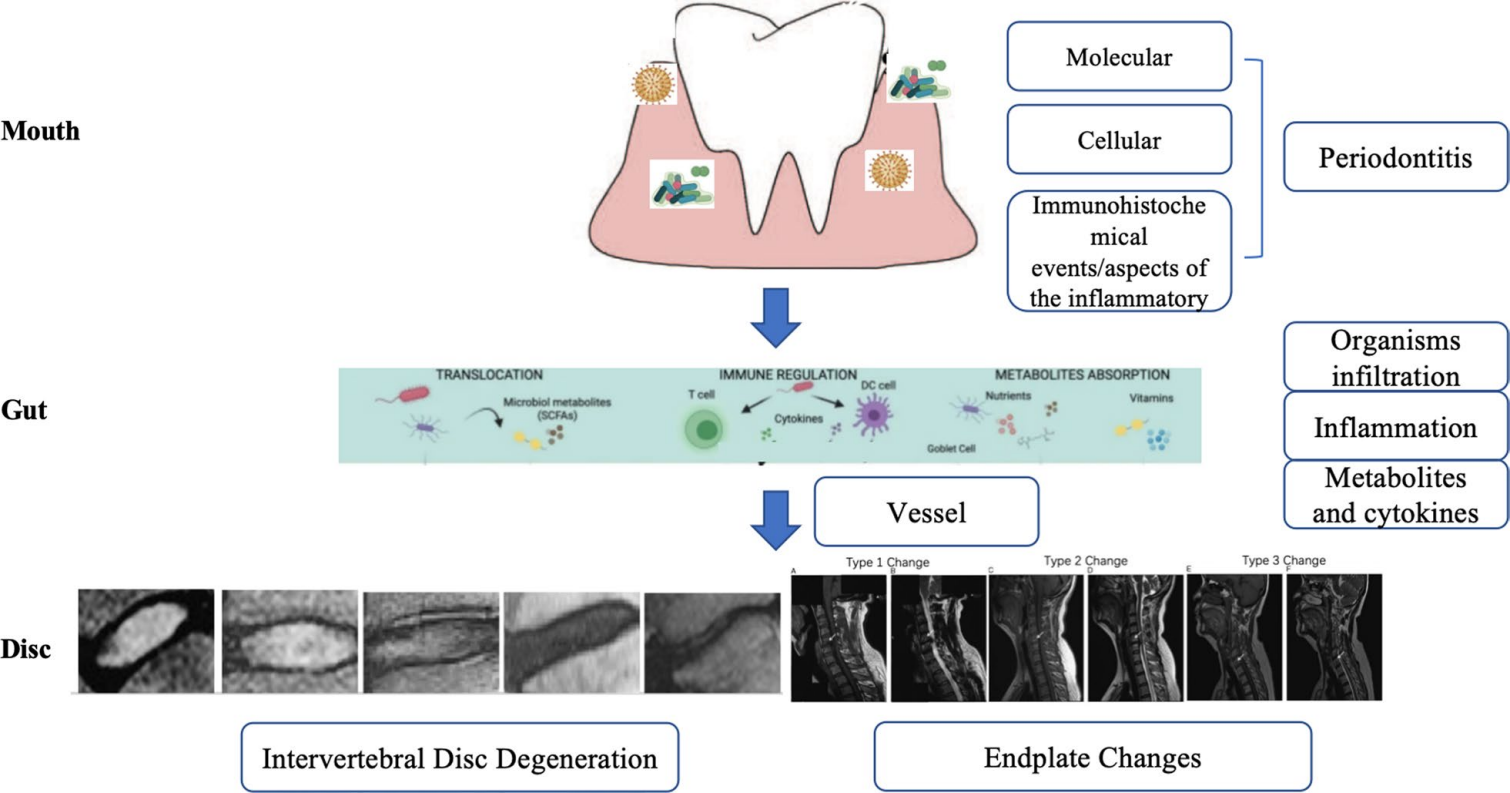
Potential mechanisms for the mouth–gut–disc axis. Oral microorganisms are the main cause of the occurrence of periodontitis. In the meanwhile, alterations in the microbiome composition in the periodontium are causing the modulation of gut microbiota translocation and composition, increasing intestinal permeability and inflammation and modifying metabolite absorption. Microorganisms, related inflammatory cytokines, and metabolites are the potential essential mechanisms for leading intervertebral disc degeneration and endplate changes

Diverse microbial communities inhabit the oral cavity, which contains about 50 species with a subset of 1,000 species [9, 10]. The dynamic and polymicrobial oral microbiome is considered one of the direct precursors to the development of periodontal health deterioration, such as periodontitis [11]. Periodontitis is thought to be driven by a feedforward loop between the microbiota and host factor, which favors the emergence and persistence of dysbiosis [12, 13]. Intriguingly, alterations in the microbiome composition in the periodontium are associated with regulating inflammation in many other diseases, like cardiovascular diseases, rheumatoid arthritis, diabetes, cancer, and chronic respiratory diseases [14–16]. Alterations in the oral microbiota promote systemic inflammation, which is thought to be the reason for the development of a metabolic syndrome in patients with periodontal diseases [17, 18]. Furthermore, previous studies have reported the detection of low-virulence organisms in degenerated cervical IVD tissue, which might play an important role in the disc structural failure process for stimulating inflammation [17, 18]. Three potential mechanisms by which the gut microbiota can induce disc structural failures are (1) bacteria infiltration across the gut epithelial barrier, (2) activation of the inflammation, and (3) modification of metabolites and cytokines absorption [8]. Taken together, these factors show the potential cascade link between the mouth–gut–disc axis and the development of disc structural failures. Oral dysbiosis may cause periodontal disease and hasten the occurrence of systematic inflammation, resulting in disc structural failures. However, the potential mechanisms underlying the mouth–gut–disc axis (Fig. 1) remain elusive. Therefore, investigating the relationship between periodontal disease and the occurrence of IDD and endplate changes in the cervical spine is of great significance.

The present prospective study investigated the relationships among periodontal disease, IDD, endplate changes, and clinical outcomes in patients with cervical degenerative disorders.

## Materials and methods

###  Study design

This prospective cohort study was approved by the Medical Research Ethics Committee of our institution. All participants agree for their demographic, clinical, and radiological data to be used in this study.

###  Participant selection

Participants aged 18 years or old adults who met the criteria to undergo open cervical spine surgery were enrolled between July 2019 and December 2021. A periodic oral evaluation was performed before surgery at our institution.

Participants with a history of spinal deformity, trauma, infection, and tumor in the cervical spine; prior cervical spine surgery; and diagnosed with systemic metabolic, severe organic, and systematic diseases were excluded from this study.

###  Demographic data

Patients’ age, gender, height, weight, body mass index (BMI), duration of neck pain, positive signs, and final diagnosis were obtained during the admission period by surgeons.

###  Periodontal examination

Periodontal parameters were recorded during the basic periodontal examination by stomatologists (DX and YZ), including the history of periodontal treatment, periodontal maintenance, history of mouthwash, the number of loose teeth, the number of remaining teeth, common causes of missing teeth, plaque index (PLI), gingival recession (GR), bleeding index (BI), and probing depth (PD, measured from the gingival margin to the bottom of the pocket in six sites, including buccal, mesiobuccal, distobuccal, lingual, distolingual, and mesiolingual, except for the third molars) [19, 20]. All participants were classified into mild, moderate, and severe periodontitis following the Center for Disease Control and Prevention in partnership with the American Academy of Periodontology (CDC-AAP) criteria (Table 1) [21]. A combination of none and mild periodontitis and moderate and severe periodontitis was referred to as the no/mild group and the moderate/severe periodontitis group in this study.

**Table 1:** Diagnosis and classification criteria of periodontitis from the Center for Disease Control and Prevention in partnership with the American Academy of Periodontology (CDC-AAP)

	Definition
Normal	No evidence of mild, moderate, or severe periodontitis
Mild periodontitis	It was defined by the presence of periodontal pockets in ≥ 2 interproximal sites with a clinical attachment level of ≥ 3 mm, in ≥ 2 interproximal sites with a probing depth (PD) ≥ 4 mm (for different teeth), or in one site with a PD ≥ 5 mm
Moderate periodontitis	Clinical attachment levels (CAL) were only measured when a periodontal pocket ≥ 3 mm was defined as the algebraic sum of PD and gingival recession (GR). It was referred as ≥ 2 interproximal sites with ≥ 4 mm CAL (not on the same tooth) or ≥ 2 interproximal sites with PD ≥ 5 mm, also not on the same tooth
Severe periodontitis	It was referred as having ≥ 2 interproximal sites with CAL ≥ 6 mm (not on the same tooth) and ≥ 1 interproximal site(s) with PD ≥ 5 mm

###  Evaluation of clinical outcomes

Three questionnaires were evaluated before the surgery once written consent was obtained from the participants. The questionnaires included the Visual Analogue Scale (VAS) for evaluating neck pain and/or radicular pain, the neck disability index (NDI) for assessing function and disability, and the Japanese Orthopedic Association score (JOA) for the assessment of cervical myelopathy.

###  Evaluation of radiological outcomes

Participants’ magnetic resonance images, consisting of sagittal T1-weight fast spin-echo (FSE), sagittal T2-weighted FSE, and axial T2-weighted FSE, were obtained using 3.0 T Trio Tim scanner (Siemens, Germany). The middle slice of T2-weighted FSE images was selected for the measurement. To reduce the potential bias, the same Apple MacBook laptop and DICOM Viewer software (Philips, Netherlands) were used for the measurement.

The Pfirrmann grade was used to assess the degeneration of IVD [5, 22]. IDD was defined as Pfirrmann grade ≥ III [5, 22]. Based on IDD classification, all the participants were allocated into the non-IDD group (-, Pfirrmann grade < III) and the IDD group (+ , Pfirrmann grade ≥ III). For the endplate changes, participants were divided into the non-endplate change group (type 0) and the endplate change group (type I, II, and III) using Modic classification [23, 24].

Cervical lordosis, range of motion (ROM), and the anterior, middle, and posterior disc height of each cervical IVD level were measured using cervical spine X-rays (such as lateral, flexion, and extension images).

###  Statistical Analysis

Continuous and dichotomous data were expressed as mean ± standard deviation (SD) and number with percentage, respectively. One-way analysis of variance (ANOVA), independent samples *t* test, and Chi-square test were used to analyze differences in features and clinical scores between no/mild and moderate/severe periodontitis groups. The normality of continuous variables was assessed. Pearson’s correlation analysis was performed to evaluate the relationship between periodontal parameters and radiological and clinical outcomes. The correlation was ranked as very strong, strong, moderate, and weak based on the following values: > 0.7, 0.5–0.7, 0.3–0.5, and < 0.3, respectively. Intra-class correlation coefficient (ICC) and their 95% confidence intervals (95% CI) were used to evaluate intra- and inter-rater reliability [25]. SPSS v24.0 (SPSS Inc., USA) was used for statistical analysis. A *P* value of < 0.05 was considered statistically significant.

## Results

###  Participants characteristics

A total of 108 consecutive patients with cervical degenerative disorders (56 males and 52 females) who underwent an anterior cervical discectomy and fusion surgery from July 2019 to December 2021 at our institution were enrolled. Of these, 68 subjects were allocated into the no/mild periodontitis group and the remaining 40 subjects were allocated into the moderate/severe periodontitis group. The mean of age, BMI, and duration of pain was 42.52 years (range, 35–63 years), 23.85 kg/m^2^, and 54.88 weeks (range, 360–20 weeks), respectively. Patients were divided based on the Pfirrmann grading system, with 38 patients in the IDD group and 70 in the non-IDD group. The mean preoperative scores of VAS neck pain, JOA, and NDI were 4.87 ± 1.08, 12.30 ± 1.77, and 25.43 ± 6.97, respectively.

###  Comparison of demographic, periodontal, clinical, and radiological data between the two periodontitis groups

No significant differences were found between no/mild and moderate/severe periodontitis groups regarding age, gender, and BMI (Table 2). The number of common causes of missing teeth (*P* = 0.005), PLI (*P* = 0.003), BI (*P* < 0.001), and PD (*P* < 0.001) significantly differed between the two groups (Table 2). No significant difference was observed between the two groups in terms of the number of periodontal treatment histories, periodontal maintenance, mouthwash histories, and remaining teeth and GR.

**Table 2:** Demographic data and periodontal parameters between No/Mild and Moderate/Severe periodontitis groups

	No/mild group	Moderate/severe group	Total	*P* value
Number of patients	68	40	108	–
Female, n (%)	30 (44.1)	22 (55)	52 (48.2)	0.436
Age (years)	46.65 ± 4.10	44.30 ± 3.54	45.52 ± 3.87	0.966
BMI (kg/m^2^)	23.72 ± 2.49	24.07 ± 3.41	23.85 ± 2.84	0.086
Number of remaining teeth	21.53 ± 4.99	20.35 ± 4.23	21.09 ± 4.72	0.380
History of periodontal treatment (yes), n (%)	18 (2.6)	0	18 (1.7)	–
Periodontal maintenance (yes), n (%)	0	0	0	
History of mouthwash (yes), n (%)	0	0	0	
Common cause of missing teeth, n (%)				
Periodontal disease	6 (8.8)	18 (45.0)	24 (22.2)	0.005^**^
Cavities	46 (67.7)	20 (50.0)	66 (61.1)	
Injury	16 (23.5)	2 (5.0)	18 (16.7)	
Plaque index (PLI)	1.50 ± 0.56	2.01 ± 0.60	1.69 ± 0.62	0.003^***^
Bleeding index (BI)	1.16 ± 0.67	2.67 ± 0.42	1.72 ± 0.94	0.000^***^
Gingival recession (GR) (mm)	1.50 ± 0.97	1.52 ± 0.76	1.51 ± 0.89	0.940
Probing depth (PD) (mm)	2.27 ± 0.22	3.56 ± 0.31	2.75 ± 0.68	0.000^***^

BMI—body mass index, n—number, mm—millimeter; continuous and dichotomous data are presented as mean ± standard deviation and number (percentage)

Significant difference ^**^ P < 0.01, ^***^ P < 0.001

The preoperative NDI was significantly lower in the no/mild periodontitis group than in the moderate/severe periodontitis group (*P* = 0.004). No significant differences were found between the two groups regarding VAS neck pain, JOA, and the duration of pain (Table 3).

**Table 3:** Clinical outcomes and radiological data between no/mild and moderate/severe periodontitis groups

	No/mild group	Moderate/severe group	Total	*P* value
VAS neck pain	5.12 ± 0.95	4.45 ± 1.19	4.87 ± 1.08	0.054
JOA	12.17 ± 1.77	12.50 ± 1.79	12.30 ± 1.77	0.973
NDI	27.41 ± 4.30	22.05 ± 9.17	25.43 ± 6.97	0.004^**^
Duration of pain (weeks)	55.13 ± 16.22	52.23 ± 14.22	54.88 ± 17.31	0.642
*Pfirrmann grade of disc degeneration*				
No degeneration	46 (67.6)	24 (60.0)	70 (64.8)	0.570
Degeneration	22 (32.4)	16 (40.0)	38 (35.2)	
*Endplate change*				
Yes	10 (14.7)	20 (50.0)	30 (27.8)	0.005^**^
No	58 (85.3)	20 (50.0)	78 (72.2)	
*ROM (°)*				
C2-3	3.57 ± 2.41	3.17 ± 2.39	3.42 ± 2.39	0.551
C3-4	3.35 ± 1.93	4.13 ± 2.03	3.64 ± 1.98	0.168
C4-5	3.71 ± 2.51	3.99 ± 2.82	3.82 ± 2.60	0.711
C5-6	4.34 ± 2.00	3.68 ± 1.91	4.07 ± 2.04	0.226
C6-7	2.99 ± 1.70	3.64 ± 2.27	3.23 ± 1.93	0.235
Cervical lordosis	16.30 ± 7.66	15.21 ± 6.59	15.89 ± 7.23	0.599
*Disc height (mm)*				
*C2-3*				
Anterior	5.39 ± 0.98	5.47 ± 1.88	5.42 ± 1.36	0.856
Middle	7.08 ± 0.81	6.97 ± 1.15	7.04 ± 0.94	0.662
Posterior	5.00 ± 0.89	5.20 ± 1.92	5.07 ± 1.35	0.612
*C3-4*				
Anterior	5.30 ± 1.15	5.35 ± 1.17	5.32 ± 1.15	0.872
Middle	6.45 ± 0.96	6.38 ± 1.21	6.42 ± 1.05	0.816
Posterior	4.63 ± 1.10	4.43 ± 1.06	4.56 ± 1.08	0.516
*C4-5*				
Anterior	5.76 ± 1.46	5.31 ± 1.21	5.59 ± 1.37	0.248
Middle	6.22 ± 1.57	5.81 ± 1.71	6.07 ± 1.62	0.374
Posterior	4.30 ± 1.17	4.48 ± 1.05	4.37 ± 1.11	0.579
*C5-6*				
Anterior	5.29 ± 1.16	5.00 ± 1.02	5.19 ± 1.11	0.348
Middle	5.96 ± 1.62	5.43 ± 1.44	5.76 ± 1.56	0.226
Posterior	4.33 ± 1.40	4.31 ± 0.91	4.32 ± 1.24	0.950
*C6-7*				
Anterior	5.48 ± 1.06	5.45 ± 1.60	5.43 ± 1.24	0.706
Middle	5.81 ± 1.64	6.10 ± 1.41	5.91 ± 1.55	0.506
Posterior	4.34 ± 1.01	4.46 ± 0.92	4.38 ± 0.97	0.660

VAS—visual analogue scale, JOA—Japanese Orthopaedic Association, NDI—neck disability index, ROM—range of motion; continuous and dichotomous data are presented as mean ± standard deviation and number (percentage)

Significant difference ^**^ *P* < 0.01

The incidence rate of cervical endplate changes was higher in the moderate/severe periodontitis group than in the no/mild periodontitis group (*P* = 0.005). No significant differences were found between the two groups in terms of the incidence rates of IDD, cervical lordosis, ROM, and disc height of each cervical IVD level (Table 3).

###  Relationship between periodontal and radiological data

No significant correlation was found between periodontal data (including PLI, GR, BI, and PD) and radiological data (including cervical lordosis, ROM, and disc height of each cervical IVD level) (Table 4).

**Table 4:** Associations between periodontal parameters and radiological changes and between periodontal parameters and clinical outcomes

	Plaque index (PLI)	Bleeding index (BI)	Gingival recession (GR) (mm)	Probing depth (PD) (mm)	Number of remaining teeth
VAS neck pain	0.035 (0.802)	− 0.220 (0.110)	0.014 (0.922)	− 0.208 (0.131)	− 0.128 (0.358)
JOA	− 0.001 (0.996)	0.168 (0.223)	0.042 (0.762)	0.057 (0.681)	− 0.197 (0.154)
NDI	− 0.337 (0.013) ^*^	− 0.426 (0.001) ^**^	− 0.124 (0.372)	− 0.346 (0.010) ^*^	0.067 (0.628)
*ROM (°)*					
C2-3	0.079 (0.430)	0.046 (0.672)	0.147 (0.290)	− 0.228 (0.097)	0.036 (0.798)
C3-4	0.253 (0.065)	0.148 (0.286)	0.040 (0.775)	0.170 (0.219)	− 0.266 (0.052)
C4-5	− 0.031 (0.826)	0.142 (0.307)	− 0.200 (0.146)	0.097 (0.486)	− 0.143 (0.302)
C5-6	− 0.106 (0.447)	− 0.226 (0.100)	− 0.185 (0.181)	0.007 (0.901)	0.103 (0.460)
C6-7	− 0.076 (0.587)	0.054 (0.700)	0.009 (0.946)	− 0.001 (0.996)	0.117 (0.398)
Cervical lordosis (°)	− 0.119 (0.393)	− 0.046 (0.740)	− 0.174 (0.207)	− 0.084 (0.545)	0.042 (0.761)
*Disc height (mm)*					
*C2-3*					
Anterior	0.236 (0.086)	0.053 (0.706)	0.115 (0.212)	− 0.261 (0.057)	− 0.111 (0.426)
Middle	− 0.145 (0.296)	− 0.008 (0.954)	0.013 (0.928)	− 0.086 (0.536)	0.114 (0.201)
Posterior	0.161 (0.103)	− 0.045 (0.747)	0.016 (0.911)	− 0.174 (0.209)	0.170 (0.218)
*C3-4*					
Anterior	0.062 (0.657)	0.175 (0.206)	0.171 (0.061)	0.078 (0.573)	0.118 (0.196)
Middle	0.059 (0.670)	0.123 (0.377)	− 0.096 (0.489)	0.078 (0.576)	− 0.200 (0.148)
Posterior	− 0.222 (0.106)	− 0.191 (0.166)	− 0.033 (0.810)	− 0.207 (0.134)	− 0.221 (0.109)
*C4-5*					
Anterior	0.150 (0.280)	− 0.164 (0.2377)	0.004 (0.974)	− 0.185 (0.180)	− 0.123 (0.375)
Middle	− 0.029 (0.837)	− 0.118 (0.396)	− 0.025 (0.859)	− 0.085 (0.540)	0.138 (0.321)
Posterior	0.075 (0.590)	0.077 (0.582)	− 0.130 (0.350)	− 0.015 (0.917)	− 0.028 (0.839)
*C5-6*					
Anterior	0.154 (0.095)	− 0.097 (0.484)	− 0.020 (0.887)	− 0.137 (0.332)	0.181 (0.189)
Middle	− 0.149 (0.284)	0.076 (0.584)	0.030 (0.829)	− 0.050 (0.719)	0.049 (0.743)
Posterior	0.019 (0.894)	0.073 (0.602)	− 0.177 (0.200)	0.084 (0.545)	0.155 (0.262)
*C6-7*					
Anterior	− 0.226 (0.101)	− 0.239 (0.082)	− 0.091 (0.513)	− 0.075 (0.591)	− 0.039 (0.780)
Middle	− 0.014 (0.921)	0.231 (0.093)	− 0.015 (0.915)	0.215 (0.091)	0.243 (0.077)
Posterior	− 0.129 (0.351)	0.107 (0.442)	− 0.028 (0.839)	0.166 (0.232)	0.023 (0.870)

VAS—visual analogue scale, JOA—Japanese Orthopaedic Association, NDI—neck disability index, ROM—range of motion, mm—millimeter; Data was presented as coefficient value (*P* value). Significant difference ^*^ *P* < 0.05, ^**^ *P* < 0.01 (Pearson’s correlation coefficient)

###  Relationship between periodontal data and clinical outcomes

A moderate negative association was found between the NDI score and periodontal parameters (PLI: *r* = − 0.337, *P* = 0.013; BI: *r* = − 0.426, *P* = 0.001; PD: *r* = − 0.346, *r* = − 0.010), but no significant differences were observed in terms VAS neck pain and JOA scores. The GR and number of remaining teeth were unrelated to preoperative clinical outcomes, such as VAS neck pain, JOA, and NDI scores (Table 4).

### Inter-rater reliability

The inter-rater reliability for periodontal data (including GR: 0.811 (0.802, 0.855) and PD: 0.824 (0.811, 0.868)) and radiological data (including ROM: 0.832 (0.812, 0.867), disc height: 0.822 (0.801, 0.878), and cervical lordosis: 0.835 (0.821, 0.877)) ranged from good to excellent.

## Discussion

This study sought to determine the relationships between the severity of periodontitis and disc structural failures in adults with cervical degenerative disorders. There are two major findings. First, individuals with severe periodontitis had a higher incidence rate of endplate changes. Second, some specific parameters in periodontitis were negatively associated with cervical disability scores. These findings provide a better understanding of the potential mechanisms underlying the mouth–gut–disc axis in the cervical spine.

### Association between microbial colonization and IDD in the cervical spine

The human microbiome plays a key role in regulating health and diseases. 16S ribosomal RNA sequencing as the most widely used polymerase chain reaction-dependent technique can be used to analyze the microbial diversity and characterize microbiota from IDD patients. A previous review strongly supports the changes in the microbial composition and metabolites, which might emerge as an important player in regulating and managing spinal pain in patients with IDD [7, 8]. Dysbiosis is defined as the imbalance in the gut microbial community, which interrupts the diversity of different microorganisms by regulating the number of bacterial communities. Some studies provided direct evidence supporting the existence of microbial colonization in patients with IDD of the cervical spine and/or neck pain [26–29]. Therefore, it is interesting to investigate the associations between low-virulence organisms and characteristics of IVDs and endplates in the cervical spine.

###  Association between microbial colonization and periodontitis

The human oral microbiome—as one of the largest microbial communities with approximately 600 bacterial species—plays a key role in balancing the equilibrium between symbiotic or pathogenic factors and the defense mechanisms of the immune system [9, 30]. Most of the organisms have been detected in the periodontal pocket in patients with periodontitis [9, 31]. A recent meta-analysis reported an association between alterations in the composition of the oral microbiome and the development of periodontitis [13]. Oral dysbiosis induces inflammatory mediators to activate the systematic inflammation status. Intriguingly, the composition of the oral microbiome has been implicated in the potential link between gut inflammation disorders and musculoskeletal system degeneration, such as the disc [32–37]. The cascade relationships among periodontitis, the occurrence of IDD, endplate changes, and clinical outcomes in adults with lumbar degenerative disorders have been previously reported [38].

###  Mouth–gut–disc axis

Although the cause of IDD is multifactorial, infection with low-virulence organisms has been considered one of the main triggers of inflammation in the degenerative process [39, 40]. Direct infectious and indirect mechano-immunological pathways theoretically accelerate tissue damage in IVDs. Notably, current studies supporting the associations between low-virulence organisms and IDD and endplate changes mainly focus on the lumbar spine [7], with a few reports on the cervical spine. Some observational studies reported *Cutibacterium acnes* as the primarily involved pathogen in disc structural failures in the cervical spine [26–29]. Recent evidence from an animal model of cervical IVD infection by *Cutibacterium acnes* showed that low-virulence organism infection of cervical IVDs can lead to degenerative changes [41]. However, the source of low-virulence organisms in the cervical spine remains unclear.

Recently, one study provided evidence for the existence of differences in the microbiome composition in healthy, degenerative, and herniated IVD [42]. In addition, overlapping in the bacterial species among IVDs, gut, and skin provides evidence for the presence of the potential gut–disc axis microbiome [42]. Alterations in the gut microbiome potentially affect IVD via direct infectious or indirect inflammatory status. The potential mouth–gut–disc axis warrants further attention. Direct delivery of organisms, immune system modifications, and metabolite formation have been listed as the three main mechanisms for the establishment of the mouth–gut–disc axis [8].

Although oral or gut microbiota is thought to be the essential trigger of inflammation in the degenerative process, the source of the low-virulence organism for the pathogenesis of disc structural failures in the cervical spine is yet to be discovered. Our study provided indirect evidence supporting the concept of the mouth–disc axis in the cervical spine. We found a higher incidence rate of endplate changes in individuals with moderate and/or severe periodontitis. A previous study reported a significant difference in the subgingival microbiome between mild and severe periodontitis groups. The microbial composition was positively correlated with the expression of systemic inflammatory markers [17]. Taken together, oral microbiome dysbiosis in severe periodontitis may induce systemic inflammation to regulate the IDD and endplate changes, which supports the hypothesis of the current study.

###  Association between periodontitis data and clinical outcomes

In theory, alterations in oral microbiome composition aggravate the severity of PLI, BI, and PD in periodontitis, which may induce local and systemic inflammatory responses for regulating bone development and the involution process. Moreover, the inflammatory state may trigger a change in the internal and external microenvironment of IVD. Furthermore, direct invasion of organisms into the IVDs and dysregulation of local and systemic inflammatory activity can stimulate the expression of inflammatory cytokines and activate immune cells, leading to IDD and endplate changes. Inflammatory cytokines and migration of immune cells can also induce the appearance of nociceptive nerve fibers in IVDs and dorsal root ganglia. Due to these cascade responses, the expression of pain-related channels was elevated for amplifying and transmitting pain signals to the dorsal root ganglia and brain as related spinal pain [43].

###  Limitations

Nonetheless, this study has several limitations. First, although this study reports indirect evidence, direct evidence of oral microbial pathogens is missing. Second, although our and previous studies supported the group classification, the group allocation has potential bias. Third, the number of patients with smoking status and diagnosis with diabetes among the study groups were not significant differences. However, the potential bias from the risk factors in the developing disc structural failures still exists. Fourth, the pathological and laboratory tests of tissue samples were not conducted. Future prospective randomized controlled studies with a larger sample size should be performed to investigate the direct relationships between oral microbiome composition and disc structural failures and clinical outcomes.

## Conclusion

In summary, the present study identified the relationship between severe periodontitis and a high incidence rate of endplate changes and poor clinical outcomes in patients with cervical degenerative disorders. The discovery of these associations provides new insights into a novel mechanism through which the alterations in the composition of the oral microbiome potentially promote disc structural failures and pain in the cervical spine.

## Data Availability

The data that support the findings of this study are available from the corresponding author, Xiaolong Chen, upon reasonable request.
